# Prevalence of venous obstructions in (recurrent) venous thromboembolism: a case-control study

**DOI:** 10.1186/s12959-020-00238-7

**Published:** 2020-09-16

**Authors:** Pascale Notten, Rob H. W. Strijkers, Irwin Toonder, Hugo ten Cate, Arina J. ten Cate-Hoek

**Affiliations:** 1 Department of Vascular Surgery, Maastricht University Medical Centre, P.O. Box 5800, Maastricht, 6202 AZ the Netherlands; 2 CARIM, Cardiovascular Research Institute Maastricht, School for Cardiovascular Diseases, Maastricht University Medical Centre, P.O. Box 616, Maastricht, 6200 MD the Netherlands; 3 Laboratory for Clinical Thrombosis and Hemostasis, Maastricht University, P.O. Box 616, Maastricht, 6200 MD The Netherlands; 4 Thrombosis Expertise Centre, Heart + Vascular Centre, Maastricht University Medical Centre, P.O. Box 5800, Maastricht, 6202 AZ the Netherlands; 5 Thrombosis Expertise Centre, Heart + Vascular Centre, Maastricht University Medical Centre, P. Debyelaan 25, Maastricht, 6229 HX the Netherlands

**Keywords:** Venous thromboembolism, Deep vein thrombosis, Recurrence, Postthrombotic syndrome, Quality of life

## Abstract

**Background:**

The role of venous obstructions as a risk factor for recurrent venous thromboembolism has never been evaluated. This study aimed to determine whether there is a difference in prevalence of venous obstructions between patients with and without recurrent venous thromboembolism. Furthermore, its influence on the development of post-thrombotic syndrome and patient-reported quality of life was assessed.

**Methods:**

This matched nested case-control study included 32 patients with recurrent venous thromboembolism (26 recurrent deep-vein thrombosis and 6 pulmonary embolism) from an existing prospective cohort of deep-vein thrombosis patients and compared them to 24 age and sex matched deep-vein thrombosis patients without recurrent venous thromboembolism. All participants received standard post-thrombotic management and underwent an additional extensive duplex ultrasonography. Post-thrombotic syndrome was assessed by the Villalta-scale and quality of life was measured using the SF36v2 and VEINES-QOL/Sym-questionnaires.

**Results:**

Venous obstruction was found in 6 patients (18.8%) with recurrent venous thromboembolism compared to 5 patients (20.8%) without recurrent venous thromboembolism (Odds ratio 0.88, 95%CI 0.23–3.30, *p* = 1.000). After a median follow-up of 60.0 months (IQR 41.3–103.5) the mean Villalta-score was 5.55 ± 3.02 versus 5.26 ± 2.63 (*p* = 0.909) and post-thrombotic syndrome developed in 20 (62.5%) versus 14 (58.3%) patients, respectively (Odds ratio 1.19, 95%CI 0.40–3.51, *p* = 0.752). If venous obstruction was present, it was mainly located in the common iliac vein (*n* = 7, 63.6%). In patients with an objectified venous obstruction the mean Villalta-score was 5.11 ± 2.80 versus 5.49 ± 2.87 in patients without venous obstruction (*p* = 0.639). Post-thrombotic syndrome developed in 6 (54.5%) versus 28 (62.2%) patients, respectively (Odds ratio 1.37, 95%CI 0.36–5.20, *p* = 0.736). No significant differences were seen regarding patient-reported quality of life between either groups.

**Conclusions:**

In this exploratory case-control study patients with recurrent venous thromboembolism did not have a higher prevalence of venous obstruction compared to patients without recurrent venous thromboembolism. The presence of recurrent venous thromboembolism or venous obstruction had no impact on the development of post-thrombotic syndrome or the patient-reported quality of life.

## Background

Following the acute phase, a substantial number of deep-vein thrombosis (DVT) patients face long-term post-thrombotic consequences. Despite optimal conservative treatment comprising of anticoagulation therapy, early mobilisation, and the use of therapeutic compression stockings, a recurrent venous thromboembolism (reVTE) will occur in about one-third of the patients within 10 years following a first DVT [1, 2] with even higher risks in case of an unprovoked event [3, 4].

Subsequently, there is an increased risk of post-thrombotic syndrome (PTS) [5]. PTS is a chronic condition characterised by a painful, swollen limb with paraesthesia, skin changes, venous claudication, and ultimately venous ulceration. It develops in 20–50% of all DVT patients [6–8] and is associated with a negative impact on the quality of life (QoL) [9] and increased health care costs [10].

According to Virchow’s triad a disturbed or turbulent blood flow is one of the factors increasing the risk of thrombus formation. Obstruction of the venous tract, which can either be due to an anatomical anomality, or an intraluminal or extraluminal obstruction [11], induces impairment of the venous outflow [12]. This may lead to an increased risk of thrombosis due to stasis. However, it remains unknown if the presence of pre-existent venous obstruction (VO, meaning the presence of central venous obstructions and/or additional anatomic anomalies) is increased in patients with reVTE compared to patients without recurrence.

Since visualisation of the venous tract in the lower abdomen and pelvis with assessment of the inferior caval vein (ICV), common iliac vein (CIV), external iliac vein (EIV), and common femoral vein (CFV) is not incorporated into the standard diagnostic work-up for DVT, detection of VO through additional imaging is usually limited to patients presenting with more severe symptomatology in the acute phase and clinically suspect of having an iliofemoral DVT. Consequently, little is known regarding the presence of VO in relation to reVTE.

The risk of reVTE in case of venous outflow obstructions may be lowered by using (long-term) anticoagulant therapy. Additionally, new treatment modalities such as venous stenting make it possible to overcome (asymptomatic) central obstructions and restore venous flow [13]. Therefore, if the presence of VO increases the risk for recurrent thrombosis, the preferred treatment strategy in these patients might switch from conservative into a more invasive treatment also influencing the duration of anticoagulant therapy.

This exploratory case-control study had the aim to assess the prevalence of VO in patients with reVTE (i.e. recurrent DVT or pulmonary embolism (PE)) compared to patients without reVTE. In addition, the association of reVTE and VO with PTS and the experienced QoL was analysed. The underlying hypothesis was that the presence of VO increases the risk of both reVTE and PTS and is associated with a reduced QoL as well.

## Methods

### Study design

This matched nested case-control study was based on a selection of patients from an existing prospective cohort of patients with a venous thromboembolism (VTE) who received treatment according to international guidelines at the department of Internal Medicine in the Maastricht University Medical Centre, the Netherlands [14]. All patients with a first-time DVT of the femoropopliteal tract or more cranial vein segments were eligible for participation. Because the assessment for the diagnosis in the acute phase was not based on an extended duplex ultrasonography (DUS), the more cranial vein segments were not routinely identified/specified in the cohort database. Patients were excluded if they were younger than 18 years of age, pregnant, known to have an active malignancy, or if the patient refused unexpected medical findings to be communicated to the patient as well as to their general practitioner. Patients who had experienced a reVTE during follow-up were invited to participate in the current study by mail. When willing, they were requested to return a signed informed consent. Subsequently, an equal number of patients without reVTE (controls) who were matched by age and sex, were selected and approached. Informed consent had to be obtained before participation.

This study has been approved by the Medical Ethical Committee of the Maastricht University Medical Centre. All authors had full access to all the data in this study.

### Clinical assessment

All participants were invited for a single study visit at the outpatient clinic during which study assessments were performed. All study visits and assessments were performed by the same physician (RS) and registered vascular technologist (IT).

### Data collection

A standardised case record form was used to gather all study data. This form included demographics, patient characteristics, disease-specific details (dates of primary and recurrent VTEs, provoked or unprovoked cause, risk factors), current post-thrombotic treatment (use of anticoagulant therapy, type of anticoagulant therapy, duration of treatment, adherence to compression therapy), clinical scores (Villalta-scale [15]) as well as results of an extended duplex ultrasonography assessment (presence of trabeculations or compression per vein segment, reflux, anatomic anomalies, and collaterals) of the affected leg.

### Patient characteristics

The cohort database ascertained by medical records were used to obtain patient characteristics such as demographics, medical history concerning VTE, and details on the reVTE. Risk factors considered to be associated with reVTE were recent surgery, trauma or immobilization, long-term travelling, inflammation, the use of oral contraceptives, pregnancy and puerperium, obesity, a family history of VTE, and thrombophilia (factor V Leiden, antithrombin deficiency, prothrombin mutation, protein C deficiency, protein S deficiency, persistently elevated factor VIII, and/or antiphospholipid antibodies). The current use of anticoagulant medication and compression therapy as well as current risk enhancing factors for reVTE were assessed during the study visit.

### Duplex

A standardised extended DUS was performed by one dedicated registered venous technologist (IT) assessing the inferior caval vein (ICV), common iliac vein (CIV), external iliac vein (EIV), common femoral vein (CFV), femoral vein (FV), deep femoral vein (DFV), and popliteal vein (PV) of the affected leg(s) in supine position. Venous outflow obstruction through the presence of anatomical anomalies, intraluminal post-thrombotic trabeculations and/or extraluminal compression was recorded for each vein segment separately. Reflux of the CFV and PV was assessed in upright position and based on a prolonged retrograde flow [16]. All DUS assessments were performed using an Esaote Spa© 2019 type MyLabAlpha with a broadband 1–8 MHz curved array probe (AC2541).

### Post-thrombotic morbidity and quality of life

The Villalta-scale [15] was recorded to assess post-thrombotic morbidity at the time of the study visit. PTS was diagnosed according to the definition by the International Society on Thrombosis and Haemostasis (ISTH) which requires a single Villalta-score of 5 or higher or the presence of a venous ulcer obtained at 6 months or more after the initial thrombotic event [17]. PTS severity was categorised into none (Villalta score 0–4), mild (5–9), moderate (10–14), or severe (≥15 or the presence of a venous ulcer). Patient reported quality of life was assessed using the generic SF36v2 [18] and disease-specific VEINES QOL/Sym [19–21] questionnaires.

### Study outcomes

The primary study outcome was the prevalence of VO in patients with reVTE compared to patients who had not developed reVTE. Secondary study outcomes regarded duplex findings, the development of PTS, and patient-reported QoL.

A reVTE comprised a recurrent DVT (reDVT) in the legs and/or PE. ReDVT was defined as an objectified DVT of the limb involving a new venous segment or a previously involved venous segment for which earlier symptomatic and imaging improvement had been obtained in a patient with at least one prior episode of DVT [22]. Results of imaging assessments were to be compared with previous assessments and reDVT was diagnosed in case of a) a new non-compressible, previously unaffected, or normalized vein segment (popliteal, femoral, iliac, or caval), b) extension of the thrombus margin, or c) an increased thrombus size [23–25]. PE was defined as the presence of complete or partial occlusion of the lung arteries in CT-pulmonary angiogram [22]. VO was defined as presence of either extraluminal compression of the CFV and/or more cranial vein segments (e.g. due to May Thurner Syndrome, adjacent anatomical structures, or a tumour), or any anatomical anomalies (e.g. agenesis, hypoplasia, aneurysms, anatomical variances and duplicates). In this study, post-thrombotic sequelae were registered separately and were defined as the presence of intraluminal trabeculations and/or synechiae.

### Statistical analysis

Analyses were performed for the primary and secondary study outcomes by comparing the group of patients with reVTE versus those without reVTE. The study was matched on age and sex; stratified analyses on these matching factors was performed. In addition, subgroup analyses were performed for the baseline characteristics as well as secondary outcomes using the comparisons: provoked versus unprovoked VTE and VO versus no VO. Descriptive statistics were performed on patient characteristics and study outcomes using Student t-tests or Mann-Whitney U-tests for continuous variables and the Chi-squared or Fisher Exact test for categorical variables. Continuous data was presented as mean ± standard deviation, categorical data was presented with absolute number and percentages or Odds ratio (OR) and their associated 95% confidence interval (95%CI). The Jonckheere-Terpstra test was performed on the Villalta scores (total score, subjective score, and objective score) for trends. A significance level of 0.05 (two-sided) or less was considered statistically significant. All analyses were performed using SPSS, version 24.

## Results

### Patient characteristics

Forty patients with reVTE that were alive and whose contact data were available were identified from the cohort database of DVT patients treated at the outpatient clinic of the Maastricht University Medical Centre [14]. Twenty-nine patients responded to our invitation to participate in this exploratory case-control study and signed the informed consent form. These 29 patients were then age and sex matched to 29 controls who had not experienced reVTE according to the data available in the database. Therefore, in total 58 patients signed informed consent and were included in the study. Information obtained during data ascertainment of the patients’ medical records and at the study visit showed that in two out of the 29 patients the recurrent event concerned an upper extremity thrombosis. Furthermore, five of the 29 matched controls for whom no reVTE was recorded in the database actually had experienced a reVTE. Hence the final study sample includes 56 patients of which 32 patients with reVTE (26 reDVT and 6 PE) and 24 patients who had not experienced a recurrent event (Table 1). Patients had a median age of 67.0 (Inter Quartile Range 57.0–71.0) years and were predominantly male (82.1%). An unprovoked cause of the first DVT was significantly more common in patients with reVTE: 23 out of 32 patients (71.9%) versus ten out of 24 patients (41.7%), (*P* = 0.03). In seven patients (six (18.8%) versus one (4.2%); *p* = 0.219) thrombophilia was known; the prevalence of elevated factor VIII (defined as > 213% [14]) was significantly higher in patients with reVTE (six (18.8%) versus none (0.0%); *p* = 0.035).

**Table 1:** Baseline characteristics

	Recurrent VTE ***N*** = 32	No recurrent VTE ***N*** = 24	***P***-value
**Age, years**	68.0 (61.3–72.0)	65.0 (45.3–70.8)	0.223
**Sex**			0.298
- Male	28 (87.5)	18 (75.0)	0.298
- Female	4 (12.5)	6 (25.0)	0.298
**Unprovoked DVT**	23 (71.9)	10 (41.7)	0.030
**Affected side initial event**			0.697
- Left	13 (40.6)	11 (45.8)	0.697
- Right	19 (59.4)	13 (54.2)	0.697
**Affected side recurrent event**			
- Ipsilateral (± pulmonary embolism)	17 (53.1)	n/a	–
- Contralateral (± pulmonary embolism)	9 (28.1)	n/a	–
- Pulmonary embolism	6 (18.8)	n/a	–
**History of pulmonary embolism**	8 (25.0)^a^	4 (16.7)^b^	0.525
**Family history of DVT**	10 (31.3)	3 (12.5)	0.122
**Antithrombotic therapy**	32 (100.0)	17 (70.8)	0.001
**Antithrombotic therapy, type**			0.071
- VKA	26 (81.3%)	16 (66.7)	0.212
- DOAC^c^	6 (18.8%)	0 (0.0)	0.035
**Elastic compression stockings, use**	19 (59.4)	3 (12.5)	< 0.001

Data are n (%) or median (IQR)

*DOAC* Direct oral anticoagulant, *DVT* Deep venous thrombosis, *LMWH* Low Molecular Weight Heparin, *n/a* Not applicable, *VKA* Vitamin K Antagonist, *VTE* Venous Thrombo-Embolism

^a^ All pulmonary embolisms were recurrent VTE which developed after the primary thrombo-embolic event. In 6 patients it presented as a solitary pulmonary embolism and in 2 patients it presented concurrent with a recurrent deep-vein thrombosis

^b^ All pulmonary embolism were concurrent with the primary thrombo-embolic event

^c^ The DOACs used were Rivaroxaban (*n* = 4), Apixaban (*n* = 1), and Dabigatran (*n* = 1)

The current use of anticoagulant therapy and compression therapy was significantly higher in the reVTE-group: 100% (32 out of 32) versus 70.8% (17 out of 24), *p* = 0.001, and 59.4% (19 out of 32) versus 12.5% (three out of 24), *p* < 0.001, respectively. Most commonly used were the VKA: 26 out of 32 (81.3%) in patients with reVTE versus 16 (66.7%) out of 24 controls. A significant difference in the use of direct oral anticoagulants was seen, being restricted to patients with reVTE: six out of 32 (18.8%) versus none of the controls (0.0%), *p* = 0.035). Indication for indefinite treatment duration was more frequent in patients with reVTE: 30 out of 32 (93.8%) versus six out of 24 (25.0%), *p* < 0.001.

### Study outcomes

There was no significant difference in the prevalence of VO between groups: six (18.8%) in patients who had experienced a recurrent event versus five (20.8%) in patients who did not; OR 0.88 (95%CI 0.23–3.30), *p* = 1.000 (Table 2). The presence of abnormalities on duplex findings such as extraluminal compression (four (12.5%) versus three (12.5%), OR 1.00 (95%CI 0.20–4.96), *p* = 1.000), intraluminal post-thrombotic sequelae (28 (87.5%) versus 18 (75.0%), OR 2.33 (95%CI 0.58–9.43), *p* = 0.298), or venous insufficiency (19 (59.4%) versus 10 (41.7%), OR 2.05 (95%CI 0.70–6.00), *p* = 0.189) did not differ either (Table 3).

**Table 2:** Details in patients with central venous obstructions and anatomic anomalies

Recurrent VTE ***N*** = 32		No recurrent VTE ***N*** = 24		Total ***N*** = 56
6 (18.8)		5 (20.8)		11 (19.6)
Anatomic anomalies				
**#1**	Duplication of the VP, fibrosis of the VF	**#1**	Aneurysm VP	
**#2**	Duplication of the VF	**#2**	Duplication and fibrosis of the VF	
Central venous obstructions				
**#3**	Extraluminal compression: CIV and EIV	**#3**	Extraluminal compression: ICVir and CIV	
**#4**	Extraluminal compression: CIV^a^	**#4**	Extraluminal compression: ICVir and CIV	
**#5**	Extraluminal compression: ICVir and CIV^b^	**#5**	Extraluminal compression: CIV^c^	
**#6**	Extraluminal compression: CIV^c^			

Data are n (%)

*ICVir* Inferior caval vein, infra renal, *CIV* Common iliac vein, *EIV* External iliac vein, *FV* Femoral vein, *PV* Popliteal vein, *VTE* Venous thrombo-embolism

None of the variables mentioned in this table showed statistical significant difference between groups

Venous obstruction is defined as either extraluminal compression (e.g. due to May-Thurner Syndrome, adjacent anatomical structures, pelvic tumour) or the presence of anatomical anomalies (e.g. agenesis, hypoplasia, aneurysms, anatomical variances, and duplications) that might negatively influence the central venous flow

^a^ Extraluminal compression caused by spondylosis

^b^ Extraluminal compression caused by the left iliac artery

^c^ Extraluminal compression caused by May Thurner Syndrome (compression by the right iliac artery)

**Table 3:** Results duplex assessment^a^

	Recurrent VTE ***N*** = 32	No recurrent VTE ***N*** = 24	Total ***N*** = 56	Odds ratio (95%CI)
**Extraluminal compression**	4 (12.5)	3 (12.5)	7 (12.5)	1.00 (0.20–4.96)
**Extraluminal compression, per vein segment**				
ICVir	1 (3.1)	2 (8.3)	3 (5.6)	0.36 (0.03–1.16)
CIV	4 (12.5)	3 (12.5)	7 (12.5)	1.00 (0.20–4.96)
EIV	1 (3.1)	0 (0.0)	1 (1.8)	2.33 (0.09–59.8)
**Post-thrombotic sequalae**^**b**^	28 (87.5)	18 (75.0)	46 (82.1)	2.33 (0.58–9.43)
**Trabeculations, per vein segment**				
ICVsr	1 (3.1)	0 (0.0)	1 (1.8)	2.33 (0.09–59.8)
ICVir	1 (3.1)	1 (4.2)	2 (3.6)	0.74 (0.04–12.5)
CIV	2 (6.3)	3 (12.5)	5 (8.9)	0.47 (0.07–3.04)
EIV	3 (9.4)	3 (12.5)	6 (10.7)	0.72 (0.13–3.95)
CFV	3 (9.4)	4 (16.7)	7 (12.5)	0.52 (0.10–2.57)
FV	18 (56.3)	9 (37.5)	27 (48.2)	2.14 (0.73–6.32)
DFV	1 (3.1)	1 (4.2)	2 (3.6)	0.74 (0.04–12.5)
PV	27 (79.4)	16 (66.7)	43 (76.8)	2.70 (0.75–9.68)
**Venous insufficiency**^**c**^	19 (59.4)	10 (41.7)	29 (51.8)	2.05 (0.70–6.00)
**Venous insufficiency, per vein segment**				
CFV	3 (9.4)	0 (0.0)	3 (5.6)	5.81 (0.29–118.1)
PV	19 (59.4)	10 (41.7)	29 (51.8)	2.05 (0.70–6.00)

Data are n (%)

None of the variables mentioned in this table showed statistical significant difference between groups

*ICVsr* Inferior caval vein, supra renal, *ICVir* Inferior caval vein, infra renal, *CIV* Common iliac vein, *EIV* External iliac vein, *CFV* Common femoral vein, *FV* Femoral vein, *DFV* Deep femoral vein, *PV* Popliteal vein, *VTE* Venous thrombo-embolism

^a^ During the standardized duplex ultrasound study assessment the presence of extraluminal compression and/or trabeculations was assessed per individual vein segment of the affected leg(s) being the ICVsr, ICVir, CIV, EIV, CFV, FV, DFV, and PV. Venous insufficiency was assessed in the CFV and PV

^b^Post-thrombotic sequelae are defined as the presence of intraluminal trabeculations or synechiae

^c^Venous insufficiency was defined as a retrograde flow longer than 1 s [16]

Compression was seen solely in the caval and iliac tract: the ICVir (one (3.1%) versus two (8.3%) respectively, *p* = 1.000), the CIV (four (12.5%) versus three (12.5%), *p* = 1.000), and EIV (one (3.1%) versus none (0.0%), *p* = 0.126). Trabeculations were most commonly seen in the popliteal (27 (79.4%) versus 16 (66.7%), *p* = 0.200) and femoral vein (18 (56.3%) versus 9 (37.5%), *p* = 0.165). In only two (6.3%) versus five (20.8%) patients all vein segments were free of anomalies (*p* = 0.642). There was no difference for any of the results whether the right or left leg was affected.

The mean Villalta score was 5.55 ± 3.02 in patients with reVTE compared to 5.26 ± 2.63 in patients without reVTE (*p* = 0.909), composed of objective (4.03 ± 3.07 versus 3.08 ± 1.74, *p* = 0.519) and subjective (1.63 ± 1.43 versus 2.22 ± 2.30, *p* = 0.512) components. No significant trends were seen in the mean total (*p* = 0.909), objective (*p* = 0.519), or subjective (*p* = 0.512) Villalta-score. PTS and PTS severity was similar between groups. There were no differences in the reported QoL according to the SF36v2, overall score (*p* = 0.493) as well as the scores per individual category (all *P* > 0.156), and the VEINES-QOL/Sym (*p* = 0.518 for the total score and *p* = 0.966 for the intrinsic score) (Table 4).

**Table 4:** Long-term treatment outcomes

	Recurrent VTE ***N*** = 32	No recurrent VTE ***N*** = 24	Total ***N*** = 56	Odds ratio (95% CI)
**Villalta score** [15]	5.55 ± 3.02	5.26 ± 2.63	5.43 ± 2.84	–
- Subjective score	1.63 ± 1.43	2.22 ± 2.30	1.87 ± 1.85	–
- Objective score	4.03 ± 3.07	3.08 ± 1.74	3.62 ± 2.60	–
**Post-Thrombotic syndrome**^**a**^ [15, 17]	20 (62.5)	14 (58.3)	34 (60.7)	1.19 (0.40–3.51)
- None (0–4)	11 (34.4)	9 (37.5)	20 (35.7)	0.87 (0.29–2.63)
- Mild (5–9)	17 (53.1)	11 (45.8)	28 (50.0)	1.34 (0.46–3.87)
- Moderate (10–14)	3 (9.4)	3 (12.5)	6 (10.7)	0.72 (0.13–3.95)
- Severe (≥15 or venous ulceration)	0 (0.0)	0 (0.0)	0 (0.0)	0.75 (0.01–39.3)
- Missing	1 (3.1)	1 (4.2)	2 (3.6)	0.74 (0.04–12.5)
**SF-36**^**b**^ **− Reported health transition**	51.7 ± 18.5	52.1 ± 14.6	51.9 ± 16.7	–
**VEINES QOL/Sym**^**c**^	49.5 ± 11.1	51.5 ± 8.2	50.4 ± 9.9	–
**VEINES QOL/Sym, intrinsic score**^**d**^	71.3 ± 14.8	72.4 ± 12.2	71.8 ± 13.6	–

Data are n (%) or mean ± SD

None of the variables mentioned in this table showed statistical significant difference between groups

*VTE* Venous thrombo-embolism

^a^ Post-thrombotic syndrome was defined according to the definition stated by the International Society of Thrombosis and Haemostasis. This definition requires a single Villalta-score ≥ 5 assessed at 6 months or more after the acute venous thrombo-embolic event [17]

^b^The SF-36 is a questionnaire aimed at the generic health-related quality of life as reported by the patients. It comprises 36 questions covering 8 different health-related dimensions: Physical functioning, Role limitations due to physical health, Role limitations due to emotional health, Energy/Fatigue, Emotional well-being, Social functioning, Bodily pain, and General health perceptions [18]

^c^The VEINES QOL/SYM is a questionnaire addressing the disease-specific self-reported quality of life in DVT patients. It entails 25 questions regarding the limitations, symptoms, and changes encountered as a result of the acute thromboembolic event. The final summarizing score is adapted to the study population [19, 20]

^d^By using the method by Bland et al. [21] the VEINES QOL/SYM summarizing score can be transformed into an intrinsic score which allows comparison to other quality of life scores

Furthermore, analyses regarding outcomes of the DUS assessment and the clinical assessments for the Villalta-score, PTS, PTS severity, and QoL were also performed for two subgroups: patients with or without an unprovoked cause for the primary DVT and patients with and without VO.

These analyses showed that patients with an unprovoked cause of the primary DVT (*n* = 33, 58.9%) did not differ from patients with a provoked primary event nor were there differences in outcomes.

VO was found in 11 (19.6%) of the 56 patients of which seven (12.5%) included extraluminal caval or iliac compression. Patients with VO were significantly younger than patients without VO (61.0 (IQR 32.0–69.0) versus 68.0 (IQR 59.5–72.0), *p* = 0.046). Apart from age there were no other differences between these groups. Based on the definition used, compression was seen only in the patients with VO: seven (63.6%) versus none (0.0%), OR 0.01 (95%CI 0.00–0.14), *p* < 0.001. No differences were seen regarding intraluminal post-thrombotic trabeculations (nine (81.8%) versus 37 (82.2%), OR 1.03 (95%CI 0.19–5.70), *p* = 1.000) or venous insufficiency (six (54.5%) versus 23 (51.1%), OR 0.87 (95%CI 0.23–3.27), *p* = 1.000). The Villalta scores did not differ between groups (Total Villalta: 5.11 ± 2.80 versus 5.49 ± 2.87, *p* = 0.639; objective Villalta: 4.00 ± 3.02 versus 3.53 ± 2.53, *p* = 0.748; and subjective Villalta score: 1.50 ± 1.51 versus 1.96 ± 1.92, *p* = 0.546. Results regarding PTS, PTS severity, and QoL were also comparable between patients with and without VO.

## Discussion

In this exploratory case-control study we did not find a difference in prevalence of VO between patients that did and patients that did not develop a recurrent thrombotic event. Moreover, no impact of the absence of either reVTE or VO was seen regarding the development of PTS or on the experienced QoL.

Also, the sub analysis comparing patients with VO to patients without VO showed no differences in post-thrombotic trabeculations or venous insufficiency on DUS assessment. This may indicate that there is no association between the presence of VO and long-term post-thrombotic intraluminal sequelae. No differences were seen for the prevalence of PTS, the severity of PTS, and QoL. However, we did find that patients with VO were younger than patients without VO, suggesting that these patients might be at risk for VTE development at an earlier age. This would be in line with the expected initial increased risk of thrombus formation under conditions of disturbed or turbulent blood flow.

However, our results do not indicate that VO increases the risk of developing reVTE. Furthermore, the presence of VO does not result in a worse clinical outcome regarding PTS and QoL in patients with recurrence. Therefore, one needs to critically consider whether DVT patients with objectified VO or patients with asymptomatic VO will benefit from prolonged anticoagulant therapy or more invasive treatments such as venous stenting.

This study has several limitations. First of all, the number of included patients was low, this may especially affect the results of the sub analyses between patients with an unprovoked versus provoked VTE and between patients with and without VO. However, although it is an exploratory study and as such unable to be conclusive, its results can be used in the discussion regarding the need for long-term anticoagulation or indications for venous stenting. We found that VO is equally prevalent in patients with or without reVTE and as such VO is not a likely game changer for recurrent risk.

Second, the characteristics of the patients included in this study may differ from those in other post-thrombotic populations and therefore our findings might not be generalisable. Remarkable, yet without difference between the groups studied, was the high percentage of male patients (82.1%) and thrombosis with a right-sided (57.1%) thrombus localisation [5, 26, 27]. Also the incidence of PTS was high (60.7%) compared to the general post-thrombotic population [6–8] and was mainly based on high objective scores.

In addition, patients with reVTE differed from their age and sex matched controls regarding several baseline characteristics known to have a role in the pathophysiology of DVT and its post-thrombotic morbidity [5, 7, 8, 28–42]. This might have influenced the clinical outcomes. For example, the higher use of compression stockings in patients with a reVTE may have limited the development of PTS and post-thrombotic signs or symptoms [33, 34]. The reason for a difference in compliance to compression therapy could not be determined based on the available data. Since all patients were treated according to international guidelines for post-thrombotic care, by definition the anticoagulant management following a reVTE differed from management following a single episode.

Nevertheless, since the influence of VO on the occurrence of reVTE had not been studied before, our study provides, to our knowledge, the first insight in the characteristics of patients with VO. Clearly defined inclusion criteria and selection from an existing cohort of patients that received standardized post-thrombotic care are some of the strengths of this study. Furthermore, all consultations and duplex assessments were performed by the same physician (RS) and a highly experienced registered vascular technologist (IT), respectively, who both had no involvement in the primary care process of these patients.

## Conclusions

In conclusion, this exploratory study suggests that the presence of VO is not related to recurrent thrombotic events, nor to the clinical outcome following reVTE. Therefore, (asymptomatic) VO does not seem to provide a basis for specific treatment such as prolonged anticoagulant treatment or stenting, as means of reducing the risk of recurrent thrombosis.

## Data Availability

The datasets generated and/or analysed during the current study are available from the corresponding author AJtC-H (arina.tencate@maastrichtuniversity.nl) on reasonable request.

## Abbreviations

CFV: Common femoral vein
CIV: Common iliac vein
DFV: Deep femoral vein
DUS: Duplex ultrasonography
DVT: Deep-vein thrombosis
EIV: External iliac vein
FV: Femoral vein
ICV: Inferior caval vein
ICVir: Inferior caval vein, infra renal
ICVsr: Inferior caval vein, supra renal
IQR: Interquartile range
ISTH: International Society on Thrombosis and Haemostasis
OR: Odds ratio
PE: Pulmonary embolism
PTS: Post-thrombotic syndrome
PV: Popliteal vein
QoL: Quality of life
reDVT: Recurrent DVT
reVTE: Recurrent venous thromboembolism
VO: Venous obstruction
VTE: Venous thromboembolism
95%CI: 95% confidence interval
